# Correlation between Exposure to Magnetic Fields and Embryonic Development in the First Trimester

**DOI:** 10.1371/journal.pone.0101050

**Published:** 2014-06-30

**Authors:** Xiu-Juan Su, Wei Yuan, Hui Tan, Xiang-Yun Liu, Dan Li, De-Kun Li, Guo-Ying Huang, Li-Wen Zhang, Mao-Hua Miao

**Affiliations:** 1 Clinical and Translational Research Center, Shanghai First Maternity and Infant Hospital, Tongji University School of Medicine, Shanghai, PR China; 2 Department of Reproductive Epidemiology and Social Science, National Population and Family Planning Key Laboratory of Contraceptive Drugs and Devices, Shanghai Institute of Planned Parenthood Research, Shanghai, PR China; 3 Department of Child and Adolescent Health, School of Public Health, Fudan University, Shanghai, PR China; 4 Department of Obstetrics and Gynecology, The Maternal and Child Hospital of Xuhui District, Shanghai, PR China; 5 Division of Research, Kaiser Foundation Research Institute, Kaiser Permanente, Oakland, California, United States of America; 6 Children's Hospital of Fudan University, Shanghai, PR China; 7 Department of Obstetrics and Gynecology, The Fifth People's Hospital of Shanghai, School of Medicine, Fudan University, Shanghai, China; National Research Council, Italy

## Abstract

**Objective:**

To explore the correlation between maternal magnetic field (MF) exposure in daily life and embryonic development.

**Methods:**

A cross-sectional study was conducted among 149 pregnant women who were seeking induced abortion of unwanted pregnancies. Participating women were asked to wear an EMDEX Lite magnetic field meter for a 24-h period to obtain MF exposure level within 4 weeks following the abortion. Embryonic bud and sac lengths were measured through B-mode ultrasound before the surgical abortion. Embryo sections were prepared and examined for histological changes, and the apoptosis status of the deciduas was examined using the TUNEL apoptosis assay.

**Results:**

Embryonic bud length was inversely associated with maternal daily MF exposure level; the association was statistically significant at the time-weighted-average and 75^th^ percentile of MF exposure levels, with coefficients of −3.09 (*P* = 0.0479) and −3.07(*P* = 0.0228), respectively. Logistic regression for examining the risk of higher MF exposure indicated that women with her 75^th^ percentile of daily MF measurements ≥0.82 mG had a 3.95-fold risk of having a fetus with a shorter embryonic bud length than those whose daily MF exposure were <0.82 mG. MF exposure was associated with a higher degree of apoptosis, but the association was not statistically significant. We failed to find a statistical correlation between MF exposure and embryonic sac length and histological changes in the first trimester.

**Conclusion:**

Prenatal MF exposure may have an adverse effect on embryonic development.

## Introduction

Humans are ubiquitously exposed to magnetic fields (MFs) from power lines, domestic appliances, computers, printers, communication devices, and medical devices. Both *in vivo* and *in vitro* studies have shown associations between MFs and adverse health effects that include increased risk of miscarriage [1]–[3], lowered sperm quality [4], and up-regulation genes involved in apoptosis [5]–[7]. However, the biological hazard of MFs remains controversial.

Among the populations that may be of interest to MF studies, pregnant women are perhaps most vulnerable because of the susceptibility of the fetus to environmental insults. Animal studies showed that pulsed magnetic fields may hinder embryonic development in vivo and alter normal neural function, at certain intensities and frequencies [8]–[11]. Based on animal studies, the entire pregnancy is sensitive to MF exposure, with the early stage of pregnancy being more susceptible [12].

Human studies on MFs and embryonic development are very limited. The rates of miscarriage have increased with increasing level of maximum MF exposure, with a threshold of approximately 16 mG [3]. It is conceivable that any abnormal reproductive outcome may be preceded by changes in the embryo. However, no studies have reported on the effect of electromagnetic field exposure on embryonic development in humans. The present study was performed to explore the association between maternal MF exposure during pregnancy and embryonic development in the first trimester in a cross-sectional study.

## Materials and Methods

### Participants

The study was conducted among pregnant women seeking induced abortion in a maternal and child health center in Shanghai, China from 2009 to 2010. The abortion was induced in this center by vacuum aspiration in the first trimester. Women were eligible for the study if (1) they were aged between 18 and 35 years; (2) they did not have known chronic diseases (hypertension, hyperlipidemia, gallbladder disease, or diabetes) or diseases of the reproductive system (uterine myoma, endometriosis, or ovarian cyst); (3) they had completed less than 12 weeks of gestation and the abortion was due to unwanted pregnancy instead of medical reasons; and (4) they were willing to wear an EMDEX Lite magnetic field meter for 24 h on a typical day within 4 weeks following the abortion. Among 515 women who seek for induced abortion, 437 were eligible and 149 (34.1%) agreed to participate in the study. To make sure that the current MF measurement would accurately reflect relevant MF exposure during early pregnancy, those who had undergone significant changes in their working and living conditions or lifestyles in the previous 6 months such as taking vacation, change of work shift, or change of residence, were not included in the present study. This project was approved by the Scientific and Ethical Committee of the Shanghai Institute of Planned Parenthood Research. All participants provided written informed consent.

### In-person interview

Information on demographic characteristics, lifestyle factors, reproductive and contraceptive history, use of electrical appliances, and occupational exposure history was collected by trained interviewers using a structured questionnaire.

### MF measurement

In this study, the EMDEX Lite magnetic field meter (Enertech Consultants, Campbell, CA, USA) was used to measure the participants' MF exposure. The meter has been widely used in epidemiologic research, although studies have shown that it may underestimate personal exposure [13]. MF levels were measured in milligauss (mG). The meter was set to collect MF measurements every 4 seconds in the frequency range of 40–1000 Hz, which is predominately associated with power-line MFs. It was programmed to show “done” instead of the MF exposure level so that participants were not aware of their MF exposure level during the measurement period. This design was implemented to avoid changes in any routine daily activities owing to the MF level display. Participants were asked to wear the EMDEX Lite meter at the waist (the nearest position to the fetus) during a typical day and to place it next to their beds during sleep. All measurements during the 24 h period were ranked based on MF levels. Percentiles were then used to evaluate the potential effect of MF level. After the measurement was completed, women were asked to rate the representativeness of their activity patterns in the measurement period compared to a typical day during the index pregnancy as “very good,” “good,” or “poor”. Among 149 participants, 19 women were excluded from the analysis because they reported poor representativeness. All the remaining women completed at least 22 h of measurement; a majority (106 women) completed 24 h of measurement.

### Embryonic development measurement

The length, width, and height of the embryonic sac and the length of the embryonic bud were examined by two radiologists using transvaginal ultrasonography which was regularly practiced in the study center. And the gynecologist could confirm the intrauterine pregnancy and the size of the embryonic sac and/or embryonic bud accordingly, which was regarded as a necessary practice before the induced abortion was implemented. In addition, these parameters were just used for evaluating the embryonic development status, instead of as indices of nonviable pregnancy in the study. The maximum values for the length, width, and height of the embryonic sac were used to examine the correlation between MF exposure during pregnancy and embryonic sac development. Embryonic sac length was obtained for all participants. The gestational age was range from 5 to 12 weeks, among them, 71 (54.6%) participants' gestational age was within 7–8 complete gestation weeks. The gestational sac is first seen at approximately 5 weeks of gestational age and yolk sac at about 6 weeks [14]. Embryonic bud could be detected logically after the day yolk sac could be detected. In this study, embryonic bud length was detected and measured for 65 (50.0%) subjects. Among them, 38 (29.2%) participants' gestation age was within 7 to 8 complete gestation weeks.

We also examined the apoptosis in deciduas, which has been reported to be greatly intensified in case of miscarriage [15]. Decidual tissue was isolated and fixed in formalin (10%) after the vacuum aspiration procedure and was then stained with hematoxylin and eosin for histological and apoptotic analysis. The specimens were examined for pathologic change under a light microscope. Terminal deoxynucleotidyl transferase dUTP nick-end labeling (TUNEL) apoptosis assay was used to detect apoptotic cells. Grayscale was then obtained through Leica QWIN3 and Image-Pro Plus tools to evaluate the apoptosis level. Higher grayscale indicates more apoptotic cells [16]. The technician was blinded to the MF level during the test procedure.

### Statistical analysis

To quantify a woman's daily MF exposure level, we used the time-weighted average (TWA), median, and 75^th^ percentile (Q3) of the 24 h MF measurements to reflect her MF exposure level. TWA was defined as the time-weighted exposure intensity. The median of a woman's daily MF exposure was defined as the MF level above which 50% of all 24 h measurement had a higher value, which corresponded to 12 h of the exposure period (50% of 24 h). The Q3 was defined as the MF level above which 25% of all 24 h measurements had a higher value.

We used a general linear regression model to examine the correlation between maternal MF exposure and embryonic development after controlling for potential confounders. The number of known potential confounders was likely limited because of: (1) a lack of association between MF exposure and many commonly known social, demographic, and behavioral factors and (2) a small number of known risk factors for embryonic development. We evaluated common sociodemographic characteristics and known risk factors for embryonic development by univariate analysis to ensure that they truly did not confound the association between maternal MF exposure and embryonic development. A cutoff of 10% change in the coefficient of exposure was then used to evaluate whether a variable should be included in the regression model [17]. Women's age, education and gestational age were included in the final model according to this rule. Because of the skewed distribution of MF exposure measurements, these variables were log transformed before they were included in the regression model. Because embryonic development status varies greatly depending on the gestational week, we conducted sensitivity analyses within a narrow spectrum of gestational age (7–8 completed weeks). We also examined the association using odds ratio by categorizing the MF level according to its median and the embryonic development parameters according to their 25^th^ or 75^th^ percentiles, as shorter embryonic sac or bud length and higher grayscale indicate adverse embryonic development.

A *P* value <0.05 was considered statistically significant. The statistical software package SAS version 9.1 (SAS Institute Inc., Cary, NC, USA) was used for data analysis.

## Results

Table 1 shows the MF exposure level according to the women's characteristics. Women with a higher education level were less likely to be exposed to higher TWA levels, and this difference was statistically significant. Participants with a history of passive smoking or vaginal bleeding during the present pregnancy had higher MF exposure levels, whereas alcohol drinkers had a lower exposure level than non-drinkers, but none of these differences were statistically significant.

**Table 1:** Characteristics of the study population in relation to parameters of MF exposure (N = 130).

Characteristic	Category	N	Median (Q1,Q3)		
			TWA	Median	Q3
Age	≤20	13	0.68 (0.50,1.25)	0.46 (0.38,0.52)	0.63(0.48,0.72)
	20–25	49	0.85 (0.55,1.78)	0.52 (0.32,0.82)	0.82 (0.57,1.67)
	25–30	47	1.00 (0.61,1.57)	0.63 (0.43,1.08)	0.97 (0.68,1.88)
	>30	21	0.89 (0.65,1.31)	0.52 (0.48,0.77)	0.97 (0.63,1.63)
Education	Junior & below	38	1.22 (0.59,2.09)^a^	0.52 (0.38,0.93)	0.93 (0.57,3.63)
	Senior to college	45	0.87 (0.55,1.57)	0.52 (0.38,1.52)	0.77 (0.57,2.03)
	College & above	47	0.79 (0.65,1.40)	0.52 (0.38,0.82)	0.82 (0.63,1.52)
Occupation	Business service^b^	82	0.85(0.54–1.40)	0.48(0.38–0.93)	0.77(0.57–1.67)
	public institution^c^	14	1.15(0.79–1.95)	0.75(0.52–1.52)	0.99(0.82–1.88)
	unemployment	18	0.82(0.51–1.85)	0.50(0.43–0.72)	0.68(0.57–1.38)
	others	16	0.86(0.66–1.60)	0.60(0.52–0.85)	0.91(0.68–2.26)
Disease history ^d^	Yes	31	1.00 (0.65,2.09)	0.52 (0.38,1.02)	0.93 (0.48,1.63)
	No	99	0.85 (0.56,1.46)	0.52 (0.43,0.97)	0.82 (0.63,1.73)
Passive smoking	Yes	80	0.88 (0.58,1.43)	0.55 (0.43,0.95)	0.91 (0.63,1.65)
	No	50	0.76 (0.57,1.78)	0.52 (0.38,0.97)	0.77 (0.52,2.13)
Alcohol drinking	Yes	50	0.73 (0.54,1.25)	0.52 (0.38,0.82)	0.68 (0.57,1.52)
	No	80	0.94 (0.61,1.92)	0.57 (0.41,0.99)	0.93 (0.63,2.23)
Marital status	Single	46	0.75 (0.54,1.45)	0.50 (0.38,0.82)	0.75 (0.57,1.67)
	Married	84	0.94 (0.59,1.72)	0.55 (0.43,0.97)	0.91 (0.63,1.70)
Vaginal bleeding	Yes	24	0.99(0.67,1.86)	0.55(0.41,0.98)	0.93(0.66,2.70)
	No	106	0.86 (0.57,1.47)	0.52 (0.38,0.97)	0.82 (0.57,1.63)
First pregnancy	Yes	28	1.05 (0.53,1.60)	0.52 (0.38,1.35)	0.88 (0.55,1.81)
	No	102	0.87 (0.59,1.46)	0.52 (0.38,0.88)	0.83 (0.63,1.67)
Having a pet	Yes	21	0.76 (0.56,1.32)	0.52 (0.43,0.77)	0.72 (0.68,1.23)
	No	109	0.89 (0.59,1.58)	0.52 (0.38,0.97)	0.88 (0.57,1.67)
Interior decoration	Yes	10	0.76 (0.54,1.25)	0.52 (0.38,0.97)	0.70 (0.48,1.27)
	No	120	0.89 (0.59,1.70)	0.52 (0.38,0.95)	0.86 (0.63,1.93)
Total		130	0.88(0.58,1.57)	0.52(0.38,0.97)	0.83(0.57,1.67)

TWA, time-weighted average; MF, magnetic field.

a ANOVA, *P*<0.05; ^b^ including receptionist in company, or waitress in restaurant or cashier in supermarket; ^c^ workers in government or management department of social affairs ministry; ^d^ Diseases include reproductive tract infections, allergies and influenza.

Table 2 shows that the TWA, median, and Q3 of women's MF exposure were all inversely associated with embryonic sac and bud lengths after adjustment for women's age, education and gestational age. However, statistically significant association was only observed for embryonic bud length when TWA and Q3 of MF exposure were considered; the coefficients were −3.09 (*P* = 0.0479) and −3.07 (*P* = 0.0228), respectively. Increasing MF levels were associated with higher apoptosis grayscale, but the association was not statistically significant. The same analyses were conducted among women with gestational ages of 7–8 completed weeks and similar results were obtained.

**Table 2:** Correlation between maternal MF exposure and embryonic development.

	Embryonic sac		Embryonic bud		Apoptosis level	
	Coefficient^a^	*P* value	Coefficient^a^	*P* value	Coefficient^a^	*P* value
TWA	−0.58	0.7820	−3.09	0.0479	11.34	0.2468
median	−1.55	0.3937	−1.42	0.2957	10.32	0.2317
Q3	−2.13	0.2411	−3.07	0.0228	12.29	0.1455

MF, magnetic field.

a Adjusted for subjects' age, education and gestational age.

To examine the association between MF level and embryonic development in terms of odds ratio, we divided the participants into 2 groups by the median of Q3 (0.82 mG) and transferred the embryonic development parameters into dichotomous variables according to their 25^th^ or 75^th^ percentiles. Table 3 shows a higher median MF level (≥0.82 mG) had a 3.95 times risk of shorter embryonic bud length (≤7 mm) compared with a lower median MF level. We failed to find a statistically significant association between MF and embryonic sac length or apoptosis.

**Table 3:** MF exposure and the risk of abnormal embryonic development.

	Maternal MF exposure		
Embryonic development	≥0.82 mG	<0.82 mG	aOR (95% CI)^a^
	N (%)	N (%)	
Embryonic sac length			
≤25^th^ percentile (20 mm)	22 (31.88)	14 (22.95)	1.56 (0.70,3.48)
>25^th^ percentile	47 (68.12)	47 (77.05)	
Embryonic bud length			
≤25^th^ percentile (7 mm)	14 (45.16)	5 (14.71)	3.95 (1.10,14.20)
>25^th^ percentile	17 (55.84)	29 (85.29)	
Apoptosis grayscale			
≥75^th^ percentile (150.828)	9 (13.04)	11 (18.03)	0.87 (0.31,2.41)
<75^th^ percentile	60 (86.96)	50 (81.97)	

a Adjusted for subjects' age, education and gestational age.

Minor histological changes in the deciduas were observed in 8 participants; these included changes in neutrophil granulocyte, hemosiderin, small thrombosis, and lymphocytes detected and nourish group of cell's vesicles, but all these changes were clinically acceptable and were not included in further statistical analysis because of the small sample size.

## Discussion

We found that increased maternal MF exposure was associated with shortened embryonic bud length. We also found that a higher MF exposure (Q3≥0.82 mG) was associated with a 3.95 times risk of shorter embryonic bud length (≤7 mm) compared with a lower MF exposure level. Increasing MF level was also associated with higher apoptosis grayscale in decidual cells, but the association was not statistically significant. Clinically significant pathological alteration of the deciduas was not observed in the present study, and its association with daily MF exposure was thus not examined.

The epidemiologic evidence on the potential adverse effects of MFs on reproductive health remains controversial and inconclusive [18]–[21]. Miscarriage was the only outcome for which there was some suggestive evidence of an association with MF exposure [1]–[3]. Women exposed to a high MF level (>16 mG) have been reported to have an increased risk of miscarriage, and a stronger association for an early miscarriage [3]. This was in line with our finding that MF exposure was associated with decreased embryonic bud length.

Numerous studies have shown the adverse effect of MF levels [9], [22]–[25]. Most studies of the association between MFs and embryonic development have been performed in chickens or rats and showed that electromagnetic fields had adverse effects on brain cells, with an increased number of apoptotic cells and degeneration of brain tissues of exposed chick or mice embryos [5], [7], [26], [27]. However, exposure levels in animal research have always been higher than those that human populations are exposed to in daily life [24], [28], [29]. This may explain the insignificant association between MF level and apoptosis in decidual cells in our study.

Our study has several strengths: (1) The participating pregnant women were in the first trimester, during which the embryo is especially vulnerable to environmental hazards [30]. (2) All participants were seeking induced abortion because of unwanted pregnancy, and thus women were less likely to change their routine daily activities as is commonly observed in planned pregnancies. (3) We used EMDEX Lite to evaluate MF exposure. This meter is small and easy to carry, and thus could objectively reflect the MF exposure level from various sources [13].

Our study also has several limitations: (1) To reduce bias caused by alteration of routine behavior, the MF meter was specifically programmed only to display the time of the day without revealing any MF exposure level. However, misclassification of MF exposure level owing to alteration in activity cannot be ruled out, and thus, the observed association may be attenuated. (2) EMDEX Lite cannot provide measurements outside the frequency range of 40–1000 Hz, which limited the generalization of the results and may produce non-differential misclassification due to the unmeasured MF exposure. (3) The embryonic development status was evaluated by embryonic bud length and sac size through B-mode ultrasound in the present study by two radiologists and thus the inter-observer variation couldn't be omitted. However, the variation is random across different MF exposure level therefore the misclassification should be nondifferential. (4) Embryonic age was calculated according to the last menstrual period, which might have been imprecise, thus resulting in misclassification of the embryonic development status. Because any misclassification of the embryonic development status was unlikely to be associated with the MF exposure level, the misclassification was nondifferential. We conducted a sensitivity analysis in a narrow spectrum of gestational age (7–8 completed weeks), in consideration of the large variation in embryonic development parameters with gestational age, and found a similar association, thus strengthening our finding. (5) We used the measurement of growth of embryo as the outcome variable, instead of any clinical indications such as miscarriage, thus we cannot draw any conclusion on the effect of MF exposure on the pregnancy outcome, and thus the clinical significance of the present study is limited. (6) We didn't collect demographic characteristics of the nonparticipants, which made the evaluation of the possible participation bias impossible. However, due to a lack of association between MF exposure and many commonly known social, demographic, and behavioral factors, it is less likely that the observed association can be explained by participation bias. (7) As a cross-sectional study, it was limited in terms of without a control group on both exposure and outcome side. Firstly, we couldn't draw any conclusion on the effect of MF exposure on the viability of the pregnancy without discriminating the miscarriage and non-miscarriage group. Secondly, it was impossible for us to find a group of subjects who were absolutely unexposed in the case of MF exposure. Although we didn't expect this would impact the internal validity of the present study, it did limit the generalizability of the study. (8) Since the surgical abortion was conducted before 12 gestational weeks, while pregnancy nausea might occur as early as 5 gestational weeks, the changed mobility of subjects who experience nausea might lead to varied representativeness of MF measurement to the true exposure level across different time period. In our study, the MF measurement was conducted within 4 weeks following the abortion when the subject's schedule was nearly the same as before pregnancy, which means the representativeness of the MF measurements may be better for those who didn't experience nausea and thus didn't have alteration of mobility. However, the possible bias caused by this poor representativeness of true exposure level may be limited due to: 1) parameters including time-weighted average, median and 75th percentile measurements were used to evaluate the exposure level in our study, which were less affected by change of mobility than any spot measurements including maximum MF exposure. 2) The proportion of time exposed to nausea is likely smaller in unwanted pregnancy compared with wanted one; since women with unwanted pregnancy tend to seek for induced abortion as soon as possible once she was aware of a possible pregnancy, as indicated by nausea or menstruation delay.

We measured MFs on one typical working day, with the assumption that activity patterns were more representative on working days. Although limited by small sample size, the present study suggests that prenatal MF exposure may adversely affect embryonic development. A large sample prospective study should be conducted to further evaluate the effect of MF exposure on embryonic development.
