# Correction: Correlation between Exposure to Magnetic Fields and Embryonic Development in the First Trimester

**DOI:** 10.1371/journal.pone.0111008

**Published:** 2014-10-08

**Authors:** The PLOS ONE Staff

There is an error in Table 1. The values in the TWA, Median and Q3 columns of the Occupation rows should have commas instead of hyphens. Please see the corrected Table 1 below.

**Table 1:** Characteristics of the study population in relation to parameters of MF exposure (N  =  130)

Characteristic	Category	N	Median (Q1,Q3)		
			TWA	Median	Q3
Age	≤20	13	0.68 (0.50,1.25)	0.46 (0.38,0.52)	0.63(0.48,0.72)
	20–25	49	0.85 (0.55,1.78)	0.52 (0.32,0.82)	0.82 (0.57,1.67)
	25–30	47	1.00 (0.61,1.57)	0.63 (0.43,1.08)	0.97 (0.68,1.88)
	>30	21	0.89 (0.65,1.31)	0.52 (0.48,0.77)	0.97 (0.63,1.63)
Education	Junior & below	38	1.22 (0.59,2.09)^a^	0.52 (0.38,0.93)	0.93 (0.57,3.63)
	Senior to college	45	0.87 (0.55,1.57)	0.52 (0.38,1.52)	0.77 (0.57,2.03)
	College & above	47	0.79 (0.65,1.40)	0.52 (0.38,0.82)	0.82 (0.63,1.52)
Occupation	Business service^b^	82	0.85(0.54,1.40)	0.48(0.38,0.93)	0.77(0.57,1.67)
	public institution^c^	14	1.15(0.79,1.95)	0.75(0.52,1.52)	0.99(0.82,1.88)
	unemployment	18	0.82(0.51,1.85)	0.50(0.43,0.72)	0.68(0.57,1.38)
	others	16	0.86(0.66,1.60)	0.60(0.52,0.85)	0.91(0.68,2.26)
Disease history ^d^	Yes	31	1.00 (0.65,2.09)	0.52 (0.38,1.02)	0.93 (0.48,1.63)
	No	99	0.85 (0.56,1.46)	0.52 (0.43,0.97)	0.82 (0.63,1.73)
Passive smoking	Yes	80	0.88 (0.58,1.43)	0.55 (0.43,0.95)	0.91 (0.63,1.65)
	No	50	0.76 (0.57,1.78)	0.52 (0.38,0.97)	0.77 (0.52,2.13)
Alcohol drinking	Yes	50	0.73 (0.54,1.25)	0.52 (0.38,0.82)	0.68 (0.57,1.52)
	No	80	0.94 (0.61,1.92)	0.57 (0.41,0.99)	0.93 (0.63,2.23)
Marital status	Single	46	0.75 (0.54,1.45)	0.50 (0.38,0.82)	0.75 (0.57,1.67)
	Married	84	0.94 (0.59,1.72)	0.55 (0.43,0.97)	0.91 (0.63,1.70)
Vaginal bleeding	Yes	24	0.99(0.67,1.86)	0.55(0.41,0.98)	0.93(0.66,2.70)
	No	106	0.86 (0.57,1.47)	0.52 (0.38,0.97)	0.82 (0.57,1.63)
First pregnancy	Yes	28	1.05 (0.53,1.60)	0.52 (0.38,1.35)	0.88 (0.55,1.81)
	No	102	0.87 (0.59,1.46)	0.52 (0.38,0.88)	0.83 (0.63,1.67)
Having a pet	Yes	21	0.76 (0.56,1.32)	0.52 (0.43,0.77)	0.72 (0.68,1.23)
	No	109	0.89 (0.59,1.58)	0.52 (0.38,0.97)	0.88 (0.57,1.67)
Interior decoration	Yes	10	0.76 (0.54,1.25)	0.52 (0.38,0.97)	0.70 (0.48,1.27)
	No	120	0.89 (0.59,1.70)	0.52 (0.38,0.95)	0.86 (0.63,1.93)
Total		130	0.88(0.58,1.57)	0.52(0.38,0.97)	0.83(0.57,1.67)

TWA, time-weighted average; MF, magnetic field

a ANOVA, *P* <0.05; ^b^ including receptionist in company, or waitress in restaurant or cashier in supermarket; ^c^ workers in government or management department of social affairs ministry; ^d^ Diseases include reproductive tract infections, allergies and influenza.
